# Use of biologics in chronic spontaneous urticaria – beyond omalizumab therapy? 

**DOI:** 10.5414/ALX02204E

**Published:** 2021-02-12

**Authors:** Martin Metz, Marcus Maurer

**Affiliations:** Charité – Universitätsmedizin Berlin, corporate member of Freie Universität Berlin, Humboldt-Universität zu Berlin, and Berlin Institute of Health; Dermatological Allergology, Allergy Center Charité, Department of Dermatology, Venereology and Allergology

**Keywords:** urticaria, angioedema, biologicals, monoclonal antibodies

## Abstract

In chronic spontaneous urticaria (CSU), itchy wheals, angioedema, or both occur regularly, often daily, and for years. An effective therapy for CSU aims at achieving complete symptom control. The current guideline for the management of CSU patients recommends non-sedative anthistamines in standard or up to 4-fold higher dosages as 1 and 2 line treatment. For most CSU patients this treatment is not sufficient; for them, the anti-IgE antibody omalizumab is the therapy of choice. Although good to very good symptom control can be achieved in most cases, there are many patients with insufficient response. For these patients, but also as an alternative to therapy with omalizumab, numerous other biologicals are currently under development. In this review, we provide an overview of possible future biologic therapies for chronic urticaria.

## Introduction 

Chronic spontaneous urticaria (CSU) is characterized by recurrent wheals, angioedema, or both over a period of more than 6 weeks without a specific trigger [49]. The average duration of CSU is mostly reported to be 5 years [12, 43, 44], but in some cases the disease may last much longer. Recent data suggest that CSU patients with angioedema, patients with coexisting chronic inducible urticaria (CINDU), or with a positive autologous skin serum test (ASST), in particular, are more likely to have a longer course of the disease [4, 19, 24, 40, 44]. CSU is one of the most common skin diseases, with an estimated point prevalence of 0.5 – 1% [33], and it causes significant impairment of quality of life in many affected individuals, comparable to that of patients with severe ischemic heart disease [36]. 

Although our understanding of the pathophysiological processes of CSU has improved significantly in recent years, some aspects of its pathogenesis remain largely unexplained. For example, the central role of mast cells (MC) in the disease is well established [50]. However, the influence of other cells such as B cells, T cells, or eosinophils is unclear, as are the details of the exact mechanisms of MC activation. Numerous findings in recent years indicate that two different autoimmune mechanisms are essentially responsible for MC activation in CSU: In the majority of patients, type I autoimmunity (“autoallergy”) is present, i.e., an auto-IgE-mediated immediate reaction against an autoantigen, an endogenous allergen. In a smaller proportion of CSU patients, type IIb autoimmunity is present, in which IgG and IgM antibodies are directed against cellular structures on MCs, for example the IgE receptor FcεRI, leading to MC activation [18, 26, 30]. The presence of such autoantibodies can be detected, for example, in a basophil histamine release assay (BHRA) [8]. Patients who have a positive BHRA (~ 20% of all CSU patients) are not only more likely to have an overall more severe and prolonged CSU, but also respond significantly worse to therapy with omalizumab, an anti-IgE monoclonal antibody, which is otherwise very successful in CSU [14]. 

As of to date, there is no curative treatment for CSU, and all currently recommended treatment options are symptomatic therapies to control and prevent urticarial symptoms. The current guideline for the treatment of CSU recommends second-generation non-sedating antihistamines in standard doses as the first step. If control is not adequate (after 2 – 4 weeks or earlier, if symptoms are intolerable), the antihistamine dose should be increased up to 4-fold. If this does not result in sufficient control, omalizumab is additionally administered [49]. For patients who do not achieve symptom relief, the current guideline recommendation is the administration of cyclosporine. Most, but by no means all, patients with CSU achieve improvement in their CSU with this therapeutic algorithm. For patients who respond only partially, not at all, or slowly to available therapies, or who cannot be optimally treated due to side effects or concomitant diseases, other safe and effective alternative or additional treatment options are needed. Currently, several such therapies are in clinical development and testing [26]. In this review, we present potential future treatments with monoclonal antibodies for patients with CSU. 

## Biologics in CSU 

**Figure 1:**
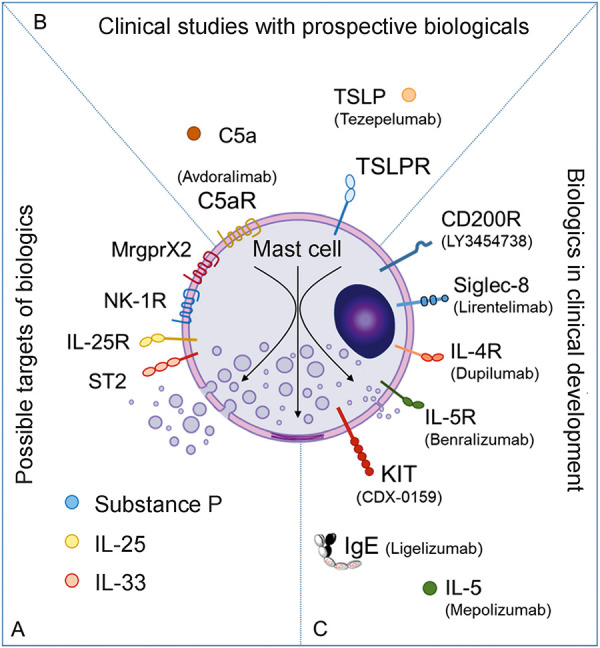
Biologics other than omalizumab for chronic spontaneous urticaria (CSU). Target structures for which biologics should be developed or existing biologics which should be clinically tested (A), drugs that are likely to be clinically tested for the indication urticaria (B), receptors and mediators for which biologics are already in clinical development for treatment of CSU, i.e., for which studies on the effectiveness and safety in patients with CSU have already been carried out or are currently being carried out (C).

MCs are the essential effector cells in urticaria; therefore, the blockade of MC activation is a promising approach in the treatment of urticaria [17]. In light of this, the current guideline for the treatment of chronic urticaria recommends omalizumab and cyclosporine as the third and fourth lines of treatment, respectively. Omalizumab prevents MC activation through IgE-mediated mechanisms, and cyclosporine can inhibit MC activation by inhibiting signal transduction. Novel biologic-based approaches in the therapy of MC-mediated diseases can be broadly divided into three groups: 1) inhibition of signals leading to MC activation, 2) activation of inhibitory receptors on MC, and 3) depletion of MC (Figure 1). In the following, we present the targets and associated monoclonal antibodies that are currently being developed or discussed for future therapy of CSU. 

## Antibodies leading to inhibition of MC-activating signals 

### Anti-IgE antibodies 

In recent years, activation of skin MC via IgE and the IgE receptor FcεRI has been shown to be a major contributor to the pathogenesis of chronic urticaria. Treatment with omalizumab, an anti-IgE antibody, is effective in CSU and is now an established therapy [1, 28, 29, 31, 32, 35, 41, 48]. With the novel anti-IgE antibody ligelizumab, there is now another monoclonal antibody that has ~ 50 times higher affinity for IgE than omalizumab. The results of a recently published multicenter, randomized, controlled phase II study show that ligelizumab is not only a highly effective therapy for CSU compared to placebo, but also has a higher rate of complete responders, i.e., patients who no longer show any symptoms of urticaria, than those treated with omalizumab [27]. Ligelizumab showed not only a very rapid and effective response, but also a longer lasting effect. At the dose of 240 mg, a recurrence of symptoms after the last administration of ligelizumab occurred on average after more than 10 weeks, whereas with omalizumab urticarial symptoms recurred after 4 weeks. Whether the better clinical efficacy of ligelizumab is due to its higher affinity for IgE or whether additional effects, for example on IgE production by B cells, are responsible for this better outcome has not yet been conclusively clarified [13]. Phase 3 trials in adults and adolescents with CSU are currently ongoing and have yet to confirm these results in a larger number of patients (NCT03580369, NCT03580356). In addition to ligelizumab, other anti-IgE biologics in development include GI-301 (GI-Innovation), a novel long-acting IgE trap-Fc fusion protein that, like omalizumab and ligelizumab, binds circulating IgE. GI-301, like ligelizumab, exhibits higher and more durable binding to IgE than omalizumab. 

### Antibodies against alarmins 

The cytokines interleukin (IL)-33, IL-25, and thymic stromal lymphopoietin (TSLP), also known as alarmins, have previously been shown to activate MCs and are thought to contribute to the pathogenesis of CSU [15, 21]. For example, it has been shown that there are significantly more cells expressing IL-33, IL-25, and TSLP in the lesional skin of CSU patients compared with that of healthy controls [15]. However, whether blockade of these cytokines actually leads to an improvement in CSU symptoms is still unknown but could represent an interesting new treatment strategy for CSU. With, for example, tezepelumab (anti-TSLP), etokimab, itepekimab, and MEDI3506 (all anti-IL-33), as well as astegolimab and GSK3772847 (both anti-ST2 (subunit of the IL-33 receptor, also called IL1RL1)), biologics are ready to test this clinically. 

### Antibodies against Th2 cytokines 

MCs express a variety of receptors, including those for the Th2 cytokines IL-4 and IL-5. It has been known for some time that both cytokines contribute to the survival of MCs and they can enhance FcεRI-mediated degranulation [37, 46]. The concentrations of IL-4 in the serum of patients with CSU are increased, and IL-4-expressing cells are found more frequently in the skin of CSU patients [7, 47], suggesting a contribution of IL-4 to the pathogenesis of CSU. Recently, a small case series has now demonstrated that dupilumab, an anti-IL-4Rα antibody, can be effective in patients with CSU [20]. The efficacy of dupilumab in urticaria is currently being investigated in several clinical trials, both in CSU and cholinergic urticaria (NCT03749135, NCT03749148, NCT04180488). 

IL-5 is another cytokine that may contribute to the pathogenesis of CSU not only through its effects on MCs but also by acting on eosinophils and basophils, which are known to be increased in the lesional skin of CSU patients. Benralizumab, an anti-IL-5 receptor antibody, as well as the anti-IL-5 antibodies mepolizumab and reslizumab have been successfully used in the treatment of individual patients with CSU and CINDU [5, 6, 22]. In addition, positive results of a smaller controlled trial with benralizumab were recently published [23]. Benralizumab and mepolizumab are currently in clinical trials to test their efficacy in CSU (NCT04612725, NCT03494881). 

### Antibodies against G protein-coupled receptors (GPCRs) 

MCs express numerous GPCRs that lead to activation and degranulation of MCs. These receptors include, for example, the complement C5a receptor (C5aR, CD88), to which the anaphylatoxin C5a binds, and the Mas-related G protein-coupled receptor X2 (MRGPRX2). Signals leading to activation of these receptors are thought to be involved in the pathogenesis of CSU. Interestingly, C5aR is selectively expressed by MCs of the skin but not by those in the lung or intestine. Since the symptoms of CSU are primarily or even exclusively manifested in the skin, this may argue for a role of C5aR. In addition, it has been shown that degranulation of MCs by autoantibodies from patients with autoimmune type IIb CSU is mediated, at least in part, by activation of C5aR [10, 16]. The efficacy of the anti-C5aR antibody avdoralimab is currently being evaluated in bullous pemphigoid, a disease in which a major role of MCs is also suspected, and other indications. The use of drugs that prevent activation of C5aR also appears to be an interesting approach for the therapy of CSU. 

Another interesting MC receptor is MRGPRX2, which, like C5aR, is preferentially expressed by skin MCs and whose expression is upregulated in the skin of patients with severe CSU [11]. MRGPRX2 is a receptor that is activated by various endogenous and exogenous substances. Endogenous agonists of the receptor include substance P, major basic protein, and eosinophil peroxidase, all of which can be found at higher concentrations in the serum of patients with chronic inflammatory skin diseases such as CSU. For substance P, a neuropeptide and agonist of both MRGPRX2 and the neurokinin 1 receptor, elevated levels are detected in the serum of CSU patients, and serum levels of substance P correlate with CSU disease activity [34, 45]. Therefore, targeted blockade of MRGPRX2 and/or its agonists, for example substance P, represents a promising mechanism to decrease MC activation in patients with CSU. 

## Antibodies that bind to inhibitory receptors on MCs 

The vast majority of receptors expressed by MCs are activating receptors, i.e., binding of corresponding ligands leads to degranulation, migration, differentiation, or proliferation of MCs. In contrast, a small group of MC receptors mediate inhibitory signals, i.e., binding of agonists to these receptors results in inhibition of MC activation, including degranulation. Two of these inhibitory MC receptors are Siglec-8 and CD200R. Antibodies directed against these receptors are currently being developed for the therapy of CSU. For example, lirentelimab, an anti-Siglec-8 monoclonal antibody, was recently shown to inhibit MC activation and lead to extensive depletion of eosinophils. Lirentelimab has been successfully tested in an open-label phase IIa pilot study in patients with omalizumab-naïve and omalizumab-refractory CSU, as well as in patients with symptomatic dermographism or cholinergic urticaria [2]. However, larger controlled studies to confirm the safety and efficacy of the drug in the treatment of CSU are still pending. Initial recently published findings on the safety of this antibody were demonstrated in a study of the therapy of eosinophilic gastritis and duodenitis [9]. 

Binding of activating antibodies to the receptor CD200R on MCs also results in inhibition of activation and degranulation of MCs [3]. The monoclonal antibody LY3454738 directed against CD200R is currently in a randomized, controlled phase 2 trial to test the efficacy of therapy in patients with CSU (NCT04159701). 

### Antibodies that deplete MCs 

MCs are among the few cells that express Kit, the receptor for stem cell factor (SCF), in the mature state. SCF is the essential factor responsible for differentiation, activation, migration, proliferation, and survival of MCs [38]. Slightly increased numbers of MCs are found in the skin of patients with CSU, which may be due to the action of SCF; moreover, SCF is a potent activator of MCs [39, 42]. Neutralization of SCF, for example by anti-SCF or anti-Kit antibodies, could reduce the number of MCs and inhibit their activation. Since any MC-mediated disease would benefit from this, it is also likely that this could be an effective therapeutic approach in CSU. Preliminary results from a phase 1 study in healthy volunteers with the anti-Kit antibody CDX-0159 indicate that treatment leads to a substantial reduction of MCs. In this study with a total of 32 healthy volunteers, a single intravenous dose-dependent administration led to an almost complete reduction of basal tryptase in the blood after only a few days. Tryptase is an MC-specific protease (only basophils still contain small amounts), and basal tryptase levels in blood (based on constitutive release by MC) correlate with the number of MC. The decrease of basal tryptase in blood after administration of CDX-0159 to levels below the detection limit therefore suggests an effective reduction of the number of MCs. In the two higher doses, there was a sustained suppression of tryptase until the end of the observation period of 71 days [25]. CDX-0159 is currently in ongoing clinical trials for CSU and CINDU. 

## Conclusion 

Until the approval of the anti-IgE antibody omalizumab for the treatment of CSU in 2014, the management of CSU patients was challenging. Because a large proportion of patients do not respond at all or inadequately to antihistamines, the advent of biologics therapy with anti-IgE represented a breakthrough in patient care. Over the years, however, it became apparent that despite the great successes with omalizumab, numerous CSU patients still could not be treated effectively. Not only to have additional therapeutic options besides omalizumab, but also to be able to treat those patients who do not respond or do not respond sufficiently to omalizumab therapy, new, safe, and effective therapeutic options are needed for CSU, but also for CINDU, for which there is still no therapy at all beyond antihistamines. 

The biologics presented here all have the potential to be equally or even more effective than omalizumab in the therapy of CSU. For some, this has already been shown in clinical trials (ligelizumab). While for some targets only the idea of a potential therapy exists so far (for example MRGPRX2), other molecules are currently already in phase 3 trials (for example ligelizumab, dupilumab). So we can be excited and optimistic about what the next years will bring for us and our patients with CSU in terms of new therapeutic options. 

## Funding 

None. 

## Conflict of interest 

M. Metz has received honoraria as a speaker and/or consultant for Amgen, Aralez, argenx, Moxie, Novartis, Roche, Sanofi, and Uriach. 

M. Maurer is or has recently been a speaker and/or consultant for and/or has received research funding from Allakos, Amgen, Aralez, ArgenX, AstraZeneca, Celldex, Centogene, CSL Behring, FAES, Genentech, GIInnovation, Innate Pharma, Kyowa Kirin, Leo Pharma, Lilly, Menarini, Moxie, Novar-tis, Roche, Sanofi/Regeneron, Third HarmonicBio, UCB, and Uriach.
